# Herbal Medicine for Behçet’s Disease: A Systematic Review and Meta-Analysis

**DOI:** 10.3390/nu13010046

**Published:** 2020-12-25

**Authors:** Ji Hee Jun, Tae Young Choi, Hye Won Lee, Lin Ang, Myeong Soo Lee

**Affiliations:** 1 Clinical Medicine Division, Korea Institute of Oriental Medicine, 1672 Yuseongdae-ro, Yuseong-gu, Daejeon 34054, Korea; zhixi04@kiom.re.kr (J.H.J.); superoung@kiom.re.kr (T.Y.C.); anglin2808@kiom.re.kr (L.A.); 2 Herbal Medicine Research Division, Korea Institute of Oriental Medicine, Daejeon 34054, Korea; hwlee@kiom.re.kr; 3 Korean Convergence Medicine, University of Science and Technology, Daejeon 34113, Korea

**Keywords:** herbal medicine, complementary and alternative medicine, Behçet’s disease, systematic review

## Abstract

Patients with Behçet’s disease often use complementary and alternative medicine for treating their symptoms, and herbal medicine is one of the options. This systematic review provides updated clinical evidence of the effectiveness of herbal medicine for the treatment of Behçet’s disease (BD). We searched eleven electronic databases from inception to March 2020. All randomized controlled trials (RCTs) or quasi-RCTs of BD treatment with herbal medicine decoctions were included. We used the Cochrane Handbook for Systematic Reviews of Interventions to assess the risk of bias and the grading of recommendations assessment, development and evaluation (GRADE) approach to assess the certainty of evidence (CoE). Albatross plot was also used to present the direction of effect observed. Eight studies were included. The risk of bias was unclear or low. The methodological quality was low or very low. Seven RCTs showed significant effects of herbal medicine on the total response rate (Risk ratio, RR 1.26, 95% CI 1.09 to 1.45, seven studies, very low CoE). Four RCTs showed favorable effects of herbal medicine on the erythrocyte sedimentation rate (ESR) and C-reactive protein (CRP) level compared with drug therapy. Herbal medicine favorably affected the ESR (MD −5.56, 95% CI −9.99 to −1.12, *p* = 0.01, I^2^ = 96%, five studies, very low CoE). However, herbal medicine did not have a superior effect on CRP. Two RCTs reported that herbal medicine significantly decreased the recurrence rate after three months of follow-up (RR 0.23, 95% CI 0.09 to 0.63, two studies, low CoE). Our findings suggest that herbal medicine is effective in treating BD. However, the included studies had a poor methodological quality and some limitations. Well-designed clinical trials with large sample sizes are needed.

## 1. Introduction

Behçet’s disease (BD), also called Behçet’s syndrome or Silk Road disease, is a multisystem chronic inflammatory disease characterized by painful mouth sores, genital ulcers, eye inflammation, and arthritis [1,2]. It frequently occurs in Turkey, Iran, Japan, and Korea. The incidence is relatively higher in East Asia than in the Mediterranean region (1–10 per 10,000 population). The incidence of BD is significantly elevated among young men [2,3].

Current BD therapies include colchicine, corticosteroids, immunosuppressive drugs, and antitumor necrosis factor-alpha (anti-TNF-alpha) agents. Western medicines help reduce symptoms and prevent complications. However, long-term treatment for BD can cause several adverse drug reactions, including osteoporosis, weight gain, fatigue, increased appetite, and increased blood pressure [4,5]. Therefore, patients are interested in complementary and alternative medicine (CAM), especially herbal medicine [6].

Clinical research studies have shown that herbal medicine may relieve BD symptoms [7]. Herbal medicine improves symptoms through cytokine modulation and significantly reduces the production of TNF-alpha, interleukin-1-beta (IL-1-beta), and interferon-gamma (INF-gamma) [8,9,10,11]. That is, herbal medicine enhances immunity by removing impurities from the body and activating blood circulation.

Recently, three systematic reviews were published on this topic [12,13,14]. Three reviews showed that herbal medicine was significantly better than drug therapy for the improvement of BD according to the clinical treatment effect, erythrocyte sedimentation rate (ESR), and C-reactive protein (CRP) level. However, the three previous reviews did not publish a transparent protocol, and they reported insufficient details pertaining to the included studies. Moreover, they are outdated.

This review aims to provide updated evidence of the efficacy and safety of herbal medicine for the treatment of BD.

## 2. Methods

### 2.1. Study Registration and Protocol Information

This review was registered at PROSPERO 2018 CRD4201808493 (Available from http://www.crd.york.ac.uk/PROSPERO/display_record.php?ID=CRD42018085493), and the protocol was published [15].

### 2.2. Data Sources

We searched for RCTs reporting the effects of herbal medicine on BD published since the inception of the databases until March 2020. We searched 11 electronic databases: PubMed, EMBASE, and CENTRAL; three Chinese databases (CNKI, Wanfang, and VIP); and five Korean databases (OASIS, DBpia, Research Information Service System (RISS), the Korean Studies Information Service System (KISS), and Korea Med). We restricted our searches to studies published in English, Chinese, and Korean. The search terms included “BD” OR “Behçet’s syndrome” OR “Behçet’s disease” AND “herbal medicine” OR “herb”. The detailed search terms are shown in Supplement 1.

### 2.3. Study Selection

#### 2.3.1. Types of Studies

All randomized controlled trials (RCTs) or quasi-RCTs comparing herbal medicine with Drug therapy were included. Master’s or doctoral theses were included. Case reports, case series, uncontrolled trials, and reviews were excluded.

#### 2.3.2. Types of Participants

Participants of both sexes and any age with clinically diagnosed BD were included. The studies had to meet the following criteria: the International Study Group (ISG) criteria [16]; the International Criteria for BD (ICBD) [17]; and the Standard for Disease in Traditional Chinese Medicine (TCM) diagnostic criteria.

#### 2.3.3. Types of Interventions and Comparison

The intervention group received only herbal medicine decoctions. Studies involving herbal medicines in pill, capsule, and powder forms were excluded. The control group received conventional medicine, no treatment, or a placebo.

#### 2.3.4. Types of Outcome Measurements

##### Primary Outcome

- The total RR: (recovery + marked improvement + improvement)/total number of cases * 100%;
- Recovery rate: clinical cure/total number of cases * 100%;
- Recurrence rate.

##### Secondary Outcome

- Changes in CRP level and the ESR in laboratory studies;
- Symptom score (oral ulcer, genital ulcer, eye inflammation, skin lesions, arthralgia);
- Adverse events (AEs).

### 2.4. Data Extraction and Risk of Bias Assessment

#### 2.4.1. Data Extraction

Two authors (J.H.J. and T.Y.C.) independently searched 11 electronic databases and read all eligible studies in full to determine the extent to which they met the eligibility criteria. Disagreements were resolved by HWL. Two authors (J.H.J. and T.Y.C.) extracted the data from the included studies. The data extraction form collected the first author, year of publication, diagnosis, sample size, duration of treatment, intervention group, control group, the main outcome, results, and AEs. Disagreements were resolved by a third author (H.W.L).

#### 2.4.2. Risk of Bias

Two evaluators (J.H.J. and T.Y.C.) assessed the studies using the risk of bias assessment tool from the Cochrane Handbook for Systematic Reviews of Interventions [18]. The following seven domains were assessed: random sequence generation, allocation concealment, blinding of participants and personnel, blinding of outcome assessment, incomplete outcome data, selective reporting, and other biases. We evaluated the risk of bias as “L” (low risk of bias), “H” (high risk of bias), and “U” (risk of bias is uncertain). Disagreements were resolved by M.S.L. We used the online version of the grading of recommendations assessment, development, and evaluation (GRADE) to assess the certainty of the evidence (CoE). The following seven categories were assessed: (1) number of studies, (2) study design, (3) risk of bias, (4) inconsistency, (5) indirectness, (6) imprecision and (7) other considerations [19].

#### 2.4.3. Data Analysis

Data analyses were performed using Review Manager (Ver. 5.3) software. For dichotomous data, we used the treatment effect as the risk ratio (RR) with a 95% confidence interval (CI). For continuous data, we present the treatment effect as the mean difference (MD) with 95% CI. The chi-squared test and Higgins I^2^ test were used to assess heterogeneity. To supplement the results for the meta-analysis of available effects, albatross plots of each included study sample size against respective *p*-values were used to provide a visual extension of effect direction for the primary and secondary outcomes using STATA/SE v.16.1 (StataCorp LLC, College Station, TX, USA).

## 3. Results

### 3.1. Description of the Included Trials

**Figure 1:**
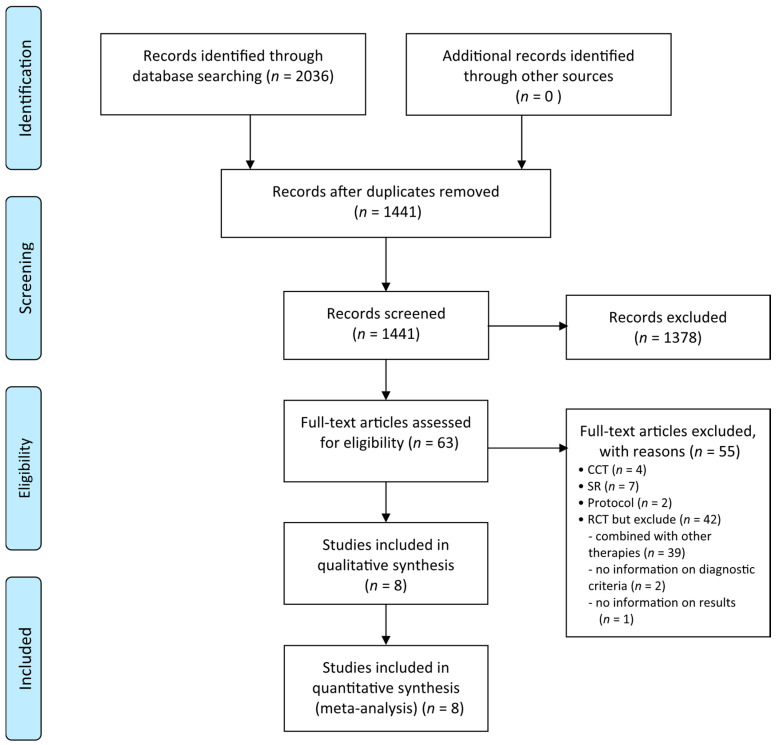
Flow chart. CCT: controlled clinical trials; RCT: randomized controlled trials; SR: systematic review.

The searches identified 2036 potentially relevant studies, of which eight [20,21,22,23,24,25,26,27] studies met our inclusion criteria (Figure 1). The key data from all included RCTs are summarized in Table 1. The RCTs published in Chinese included three master theses. The sample size ranged from 30 to 180. The duration of treatment ranged from three weeks to three months. The included studies used different disease criteria. Five RCTs [21,22,24,26,27] diagnosed BD according to the 1989 ISG criteria, two RCTs [20,23] used the 1989 ISG criteria plus the Standard for Disease in Traditional Chinese Medicine diagnostic criteria, and one RCT [25] used the 2005 ICBD criteria plus the Standard for Disease in Traditional Chinese Medicine diagnostic criteria.

For the control group treatment, three RCTs [20,21,22] used prednisone, two RCTs [23,24] used thalidomide, and three RCTs used prednisone plus thalidomide [25], loxoprofen sodium plus thalidomide [26], and interferon α-2b injection [27]. Seven RCTs used oral administration, and another RCT used injections.

The prescriptions used in the intervention group were different. The constituents of the herbal medicines used in each included study are listed in detail in Table 2. There were 8 prescriptions collected, among which 3 were set prescriptions [20,22,26], 2 were modified set prescriptions [24,25], 2 were pattern identification (PI) prescriptions [23,27], and 1 prescription was formulated based on personal experience [21]. There were 72 herbs in total. The most commonly used herbs for BD were Glycyrrhizae Radix et Rhizoma, Angelicae Sinensis Radix, Paeoniae Radix Alba, Asparagi Radix, and Glycyrrhizae Radix et Rhizoma Praeparata.

### 3.2. Risk of Bias

**Figure 2:**
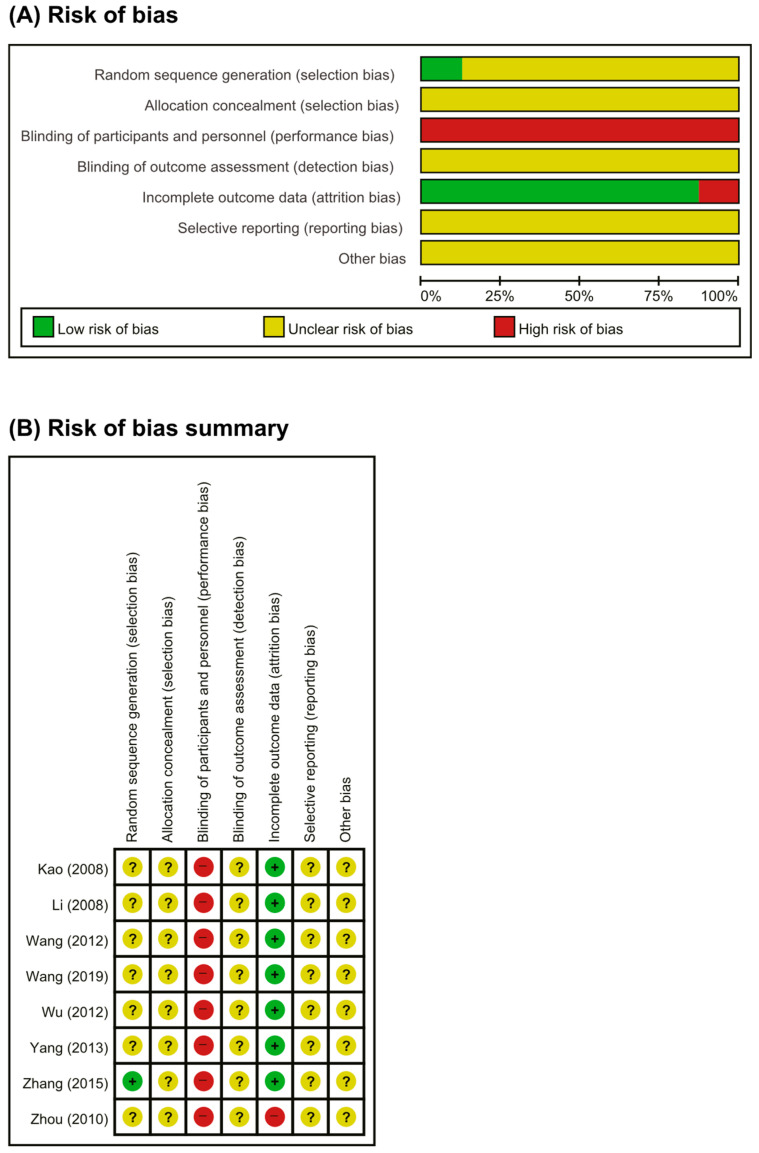
Risk of bias. (**A**) Risks of bias of graph: review authors’ judgments about each item’s risk of bias item presented as percentages across all included studies. (**B**) Risks of bias summary: review authors’ judgments about each item’s risk of bias for each included study. +: low risk of bias; −: high risk of bias; ?: unclear.

The risk of bias is presented in Figure 2. The risk of bias was assessed using the Cochrane risk of bias tool. Only one RCT [25] used the random number method for random sequence generation. Seven RCTs [20,21,22,23,24,26,27] did not report the random sequence generation method. Among the eight included RCTs [20,21,22,23,24,25,26,27], herbal medicine decoctions, and drug therapy were compared; thus, blinding could not be applied to participants and personnel. None of the RCTs described the method of allocation concealment or blinding of outcome measurement. One RCT [22] did not provide the reasons for patient drop-out and withdrawal. None of the RCTs published or registered their protocol, and they all had an unclear risk of bias with regard to selective outcome reporting.

### 3.3. Certainty of Evidence

The CoE for each outcome was either low or very low (Table 3).

### 3.4. Outcome Measurements

#### 3.4.1. Primary Outcomes

##### Total Response Rate

**Figure 3:**
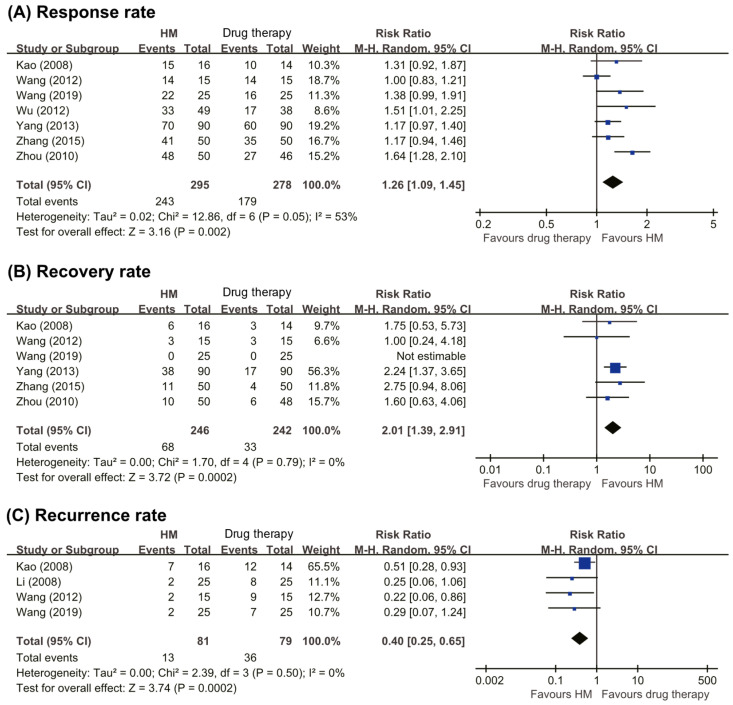
Forest plots of (**A**) total response rate, (**B**) recovery rate, and (**C**) recurrence rate. HM: herbal medicine.

Seven RCTs [20,22,23,24,25,26,27] compared the effect of herbal medicine with that of drug therapies. One RCT [22] showed favorable effects of herbal medicine on the total RR compared with drug therapies, and the other six RCTs [20,23,24,25,26,27] reported equivalent effects. The meta-analysis showed favorable effects of herbal medicine on the total response rate (RR 1.26, 95% CI 1.09 to 1.45, seven trials, *n* = 573, *p* < 0.002, I^2^ = 53%, Figure 3A).

##### Recovery Rate

Six RCTs [20,22,23,24,25,26] compared the effect of herbal medicine with that of drug therapies. One RCT [24] reported that no patients recovered in either group, while another RCT [26] reported that herbal medicine was a more effective therapy. The remaining four RCTs [20,22,23,25] showed equivalent effects in the two groups. The meta-analysis showed favorable effects of herbal medicine on the patient recovery rate (RR 2.01, 95% CI 1.39 to 2.91, six trials, *n* = 488, *p* < 0.0002, I^2^ = 0%, Figure 3B).

##### Recurrence Rate

Four RCTs [20,21,23,24] compared the recurrence rates between patients taking herbal medicine and drug therapies. Three RCTs reported that herbal medicine lowered the recurrence rate compared with conventional drugs [20,21,23,24], while one RCT found recurrence rates between the two groups after 12 months of follow-up [24]. The meta-analysis showed favorable effects of herbal medicine on the recurrence rate (RR 0.40, 95% CI 0.25 to 0.65, four trials, *n* = 160, *p* = 0.0002, I^2^ = 0%, Figure 3C).

#### 3.4.2. Secondary Outcomes

##### ESR and CRP

**Figure 4:**
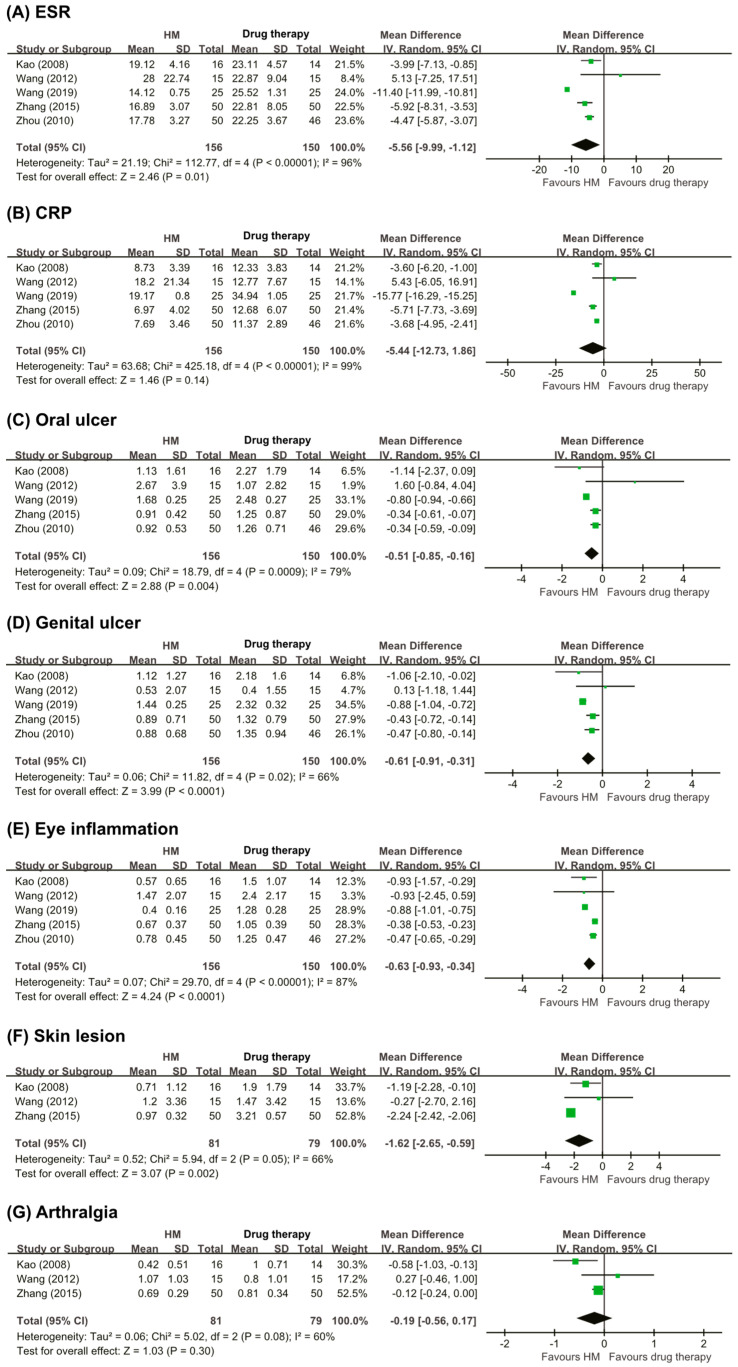
Forest plots of symptoms score (**A**) ESR, (**B**) CRP, (**C**) oral ulcer, (**D**) genital ulcer, (**E**) Eye inflammation, (**F**) skin lesions, and (**G**) arthralgia. CRP: C-reactive protein; ESR: erythrocyte sedimentation rate; HM: herbal medicine.

Five RCTs [20,22,23,24,25] compared the effects of herbal medicine and drug therapies on the ESR and CRP levels. Four RCTs [20,22,24,25] showed favorable effects of herbal medicine compared to drug therapies, and one RCT [23] showed inferior effects of herbal medicine. The meta-analysis showed a favorable effect of herbal medicine on the ESR (MD −5.56, 95% CI −9.99 to −1.12, five trials, *n* = 306, *p* = 0.01, I^2^ = 96%, Figure 4A), but failed to do so regard to the CRP level (MD −5.44 95% CI −12.73 to 1.86, five trials, *n* = 306, *p* = 0.14, I^2^ = 99%, Figure 4B).

##### Symptom Score

*Oral Ulcers*

Six RCTs [20,21,22,23,24,25] assessed the symptom score for oral ulcers. Three RCTs [22,24,25] reported superior effects of herbal medicine compared with drug therapies, while the other three RCTs [20,21,23] showed inferior results. Only five RCTs [20,22,23,24,25] were applicable for meta-analysis. The results of the meta-analysis showed a superior effect of herbal medicine to that of drug therapy on the symptom score for oral ulcers (MD −0.51, 95% CI −0.85 to −0.16, five trials, *n* = 306, *p* = 0.004, I^2^ = 79%, Figure 4C).

*Genital Ulcers*

Six RCTs [20,21,22,23,24,25] assessed the symptom score for genital ulcers. Three RCTs [22,24,25] reported that the effects of herbal medicine were superior to those of drug therapies; however, three RCTs showed inferior results. Only five RCTs [20,22,23,24,25] were included in the meta-analysis. The result of the meta-analysis showed a greater effect of herbal medicine than of drug therapies on the symptom score for genital ulcers (MD −0.61, 95% CI −0.91 to −0.31, five trials, *n* = 306, *p* < 0.0001, I^2^ = 66%, Figure 4D).

*Eye Inflammation*

Six RCTs [20,21,22,23,24,25] reported the symptom scores for eye inflammation. Three RCTs [20,22,25] reported that herbal medicine had superior effects to those of drug therapy, while three RCTs showed [21,23,24] similar effects. Only five RCTs [20,22,23,24,25] were included in the meta-analysis. The result of the meta-analysis showed favorable effects of herbal medicine with regard to reducing eye inflammation compared with drug therapy (MD −0.63, 95% CI −0.93 to −0.34, five trials, *n* = 306, *p* < 0.0001, I^2^ = 87%, Figure 4E).

*Skin Lesions*

Four RCTs [20,21,23,25] assessed the symptom scores for skin lesions. Two RCTs [20,25] reported that the effects of herbal medicine were superior to those of drug therapies, while two RCTs [21,23] showed equivalent effects between the intervention group and the control group. Only three RCTs [20,23,25] were applicable for meta-analysis. The result of the meta-analysis showed a favorable effect of herbal medicine with regard to reducing skin lesions (MD −1.62, CI −2.65 to −0.59, three trials, *n* = 160, *p* = 0.002, I^2^ = 66%, Figure 4F).

*Arthralgia*

Four RCTs [20,21,23,25] reported the symptom scores for arthralgia. One RCT [20] reported that the effects of herbal medicine were superior to those of drug therapies, while three RCTs [21,23,25] showed equivalent effects between the two groups. Only three RCTs [20,23,25] were applicable for meta-analysis. The results of the meta-analysis showed no significant difference between the two groups (MD −0.19, 95% CI −0.56 to 0.17, three trials, *n* = 160, *p* = 0.30, I^2^ = 60%, Figure 4G).

*AEs*

Three RCTs [21,23,27] reported Es, whereas five RCTs [20,22,24,25,26] did not report AEs (Table 1). One RCT [27] did not report the detailed symptoms of AEs. Two RCTs [20,23] reported Es in the intervention group, including diarrhea. The other RCT [23] reported AEs in the control group, including insomnia, dizziness, and constipation.

### 3.5. Albatross Plot

**Figure 5:**
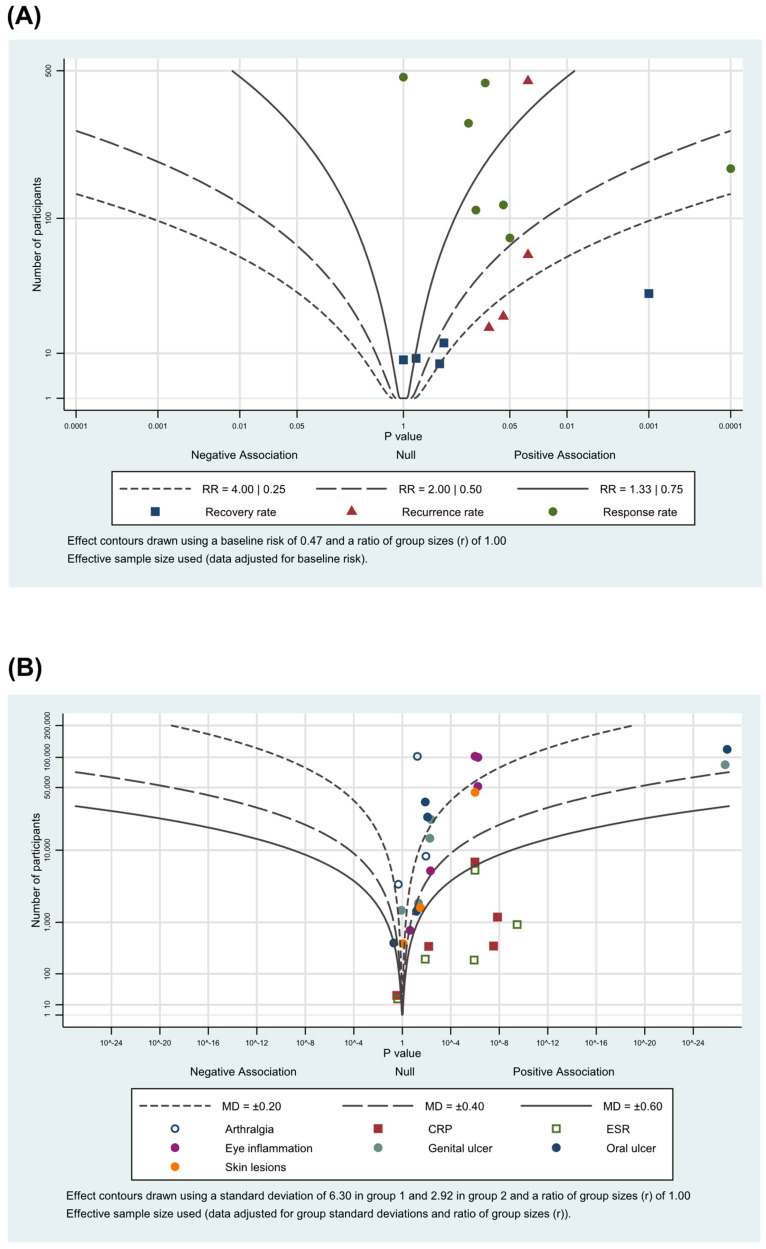
Albatross plot for (**A**) primary outcomes; (**B**) secondary outcomes.

The albatross plots for the included studies are shown in Figure 5. Albatross plots were performed by illustrating contours that showed the effect direction and effect sizes range using *p*-values and given sample sizes. Different plotting colors correspond to the outcome subgroups. Looking at the albatross plot for dichotomous data (Figure 5A), the points scattered across different contour lines. However, most of the points clustered to the right side of the plot, showing that herbal medicine was more favorable for the treatment of BD. For the albatross plot of continuous data (Figure 5B), the points were less scattered. Although the points were clustered to the right, many points were positioned around the null line, implicating non-significant effects. Both albatross plots had points that were completely isolated, reflecting the possibility of sampling error.

## 4. Discussion

### 4.1. Summary of the Main Results

The aim of this systematic review was to evaluate the effectiveness and safety of herbal medicine for the treatment of BD. Eight studies [20,21,22,23,24,25,26,27] evaluating the effect of herbal medicine on BD showed the following results. Herbal medicine reduces the ESR, reduces the symptom scores (oral ulcers, genital ulcers, eye inflammation, skin lesions), decreases the recurrence rate and improves the total clinical effective rate, although some studies have not provided evidence of the superiority of herbal medicine in terms of the symptom score for arthralgia and the CRP. Three RCTs [21,23,27] reported Es, but these Es were generally mild, and the patients spontaneously recovered. Overall, the results showed that herbal medicine decoctions might be useful in the treatment of BD.

### 4.2. Overall, Completeness and Applicability of the Evidence

This review shows that herbal medicine can be used to improve clinical symptoms in BD patients and that there are fewer Es associated with its use than with drug therapies. Despite the positive results, the included studies had small sample sizes and generally poor methodological quality; furthermore, they were too heterogeneous to allow any firm conclusions to be drawn regarding the different types of herbal prescriptions.

### 4.3. Quality of the Evidence

The CoE was low and very low for all outcomes (Table 3). Among the included RCTs, none reported the randomization methods, allocation concealment, or blinding of information. They did not publish their protocols, and it was not clear whether the planned result indicators were reported accurately. Therefore, they were downgraded one level in the risk of bias domain. The heterogeneity was substantial; thus, they were downgraded one or two levels. The included studies were PICO (patient, intervention, comparison, outcomes) studies, and it was determined that there was insufficient direct evidence of an effect. All outcomes had wide CIs that crossed the assumed threshold of the minimal clinically important difference; thus, they were downgraded one level for imprecision. Furthermore, the number of trials and total sample sizes included in our analysis was not sufficient to enable us to draw firm conclusions.

### 4.4. Potential Biases in the Review Process

This review has several limitations. First, the included studies used different herbal prescriptions, the effectiveness of which for the treatment of BD was not well known. Therefore, future studies should analyze studies using similar herbal prescriptions. Second, the evidence of improvements in symptoms varied according to the herbal decoction, possibly due to the varying compositions and dosages of the herbs. This review shows that herbal medicine has effects that are superior to those of drug therapy according to the composition of herbs, but the effects of dosages were unclear. Therefore, future studies should focus on the detailed composition and dosages of herbs. Third, all included studies were conducted in China, where no negative studies have been reported [28]. Furthermore, the albatross plots also showed scattered points across contour lines, with a few points being isolated from the other point clusters. As the sample sizes of the included studies were relatively small, this would likely reflect possible sampling bias.

### 4.5. Agreements and Disagreements with Other Studies or Review

We found three previous systematic reviews [12,13,14] on the use of herbal medicine for BD. These studies reported that herbal medicine was better than drug therapy for the treatment of BD. We identified two new RCTs [24,25] and extracted evidence from them. The results and evidence levels were similar to those of the studies included in the three previous systematic reviews. Moreover, the authors of those reviews expressed concern regarding the small sample sizes and the poor quality of the included studies. Future well-designed RCTs with large sample sizes are thus warranted.

### 4.6. Potential Mechanism of Action

In spite of the comparative absence of compelling evidence toward herbal medicines for BD, the potential features that may be related point towards benefits. These properties include anti-inflammation, immunoregulation, and antioxidation with chronic autoimmune disease and the studies focusing on the fusion of medicinal plants and cytokine activity effects. Since the disparity in the expression of innate immunity-related cytokines cannot only play a crucial role in BD pathogenesis but can also be pivotal in the level of severity of the disease [29]. Further, the disease activity score and clinical activity index may also be influenced by the levels of anti-inflammatory cytokines [30,31]. The herbal prescriptions mostly used Glycyrrhizae Radix et Rhizoma and Angelicae Sinensis Radix, which have anti-inflammatory effects [32,33]. The biological of herbal medicines linked with BD were not focused on in the present review. However, herbal medicines’ properties used to treat BD must be researched further.

The therapeutic effects of herbal medicine possibly rely on the obtainability and quantity of the different components in the production. The applicable data are sometimes not included in many publications. The daily prescribed quantity of the trials included in the study is diverse across the included studies-in their condition severity and traditional diagnosis type. However, no research has been conducted on the prime dose to reduce BD symptoms. In the present study, when we analyzed the result direction and dosage, treatment time, dosage and time, and the type of results, no direct links with dose and the treatment time for relevant changes of various results. The variety in trials does not present clear relations. Different herbal medicines were compared and examined with drug therapies using several methods. The contrasts in significance may result from the type of herbal medicines and dosage in treatments. The quantity and frequency of herbal medicines utilized in the trials included in the study may be inadequate to create a noteworthy effect in biochemical variables. Thus, it is required to conduct studies ranging in doses and comparing a variety of herbal medicines to various outcomes to answer such questions.

### 4.7. Implications for Nutrients

The applicability of the present review could be questioned in the fields of nutrients. Herbal materials are derived from plants, and many such substances are part of both food supplements and nutraceuticals as well as medicinal products [34,35,36,37]. In the US, regulatory bodies such as FDA governing plant-based medicines usually regard them as dietary supplements [35]. Dietary recommendations refer to herb usage as an outstanding source of antioxidants in Australia [34,38]. Furthermore, in Traditional East Asian Medicine, such medicines are consumed in the form of herbal tea or supplemental food. With the increase of the presence of herbs in diets owing to their health advantages, utilization of herbal medicines for BD may be possible in the capacity of a herbal supplement, in addition to the main diet containing functional foods or nutraceuticals per respective regulatory systems across countries [35,39,40,41]. However, such supplements for BD should ensure avoidance of any side effects by undergoing quality testing and safety protocols taken for dietary supplements and functional foods.

### 4.8. Implications for Practice

BD is a chronic inflammatory disease in which ulceration occurs repeatedly. Herbal medicine is associated with a lower rate of recurrence and is relatively safer than drug therapies. It appears useful in clinical practice. However, the included studies had only short-term treatment periods and used various forms of prescriptions. Thus, long-term clinical research and standardized prescriptions should be implemented in future studies.

### 4.9. Implications for Research

This review has several limitations with regard to the research process. First, the risk of bias in this review was unclear. The majority of trials did not report randomization procedures, and all of them lacked information on blinding. None of them reported the randomization and allocation methods, published their protocols, or registered at PROSPERO. Thus, future studies should be described in detail or registered at PROSPERO. Second, rigorous RCTs should be carried out to analyze the effectiveness of herbal medicine for the treatment of BD. Adequate data on the clinical outcomes of BD treated with herbal medicine could guide clinical decision-making. Future studies should be comprehensively reported according to the CONSORT reporting guidelines [42].

## 5. Conclusions

This review showed that herbal medicine decoctions might be useful in the treatment of BD. However, the quality of the current evidence was low, the small effect size reduced the clinical significance, and the small number of rigorous studies prevented us from drawing firm conclusions. Well-designed RCTs are needed to determine whether herbal medicine is a viable option for the treatment of BD.

## Figures and Tables

**Table 1:** Summary of randomized controlled trials of herbal medicine for Behçet’s disease.

First Author (Year) [Ref]	Sample Size (Randomized/Analyzed) Mean Age (Year) Disease Duration (Year) Diagnosis	Intervention Group (Regimens)	Control Group (Regimens)	Main Outcome	Results	Adverse Effect
Kao (2008) [20]	30/30 A: 30.2; B: 29.8 A: 7.6; B: 8.1 1989 ISG; 1994 Standards TCM diagnosis criteria	(A) HM (Yiqi Tuodu decoction, 2 times daily for 2 months, *n* = 16)	(B) Drug therapy (prednisone 10 mg, 2 times daily for 2 months, *n* = 14)	(1) Response rate (2) Recover rate (3) ESR (4) CRP (5) Symptom score (6) Recurrence rate (n.r.)	(1) RR 1.31 [0.92, 1.87], NS (2) RR 1.75 [0.53, 5.73], NS (3) MD −3.99 [−7.13, −0.85], *p* = 0.01 (4) MD −3.60 [−6.20, −1.00], *p* = 0.007 (5) Oral ulcer: MD −1.14 [−2.37, 0.09], NS; genital ulcer: MD −1.06 [−2.10, 0.02], *p* = 0.05; eye inflammation: MD −0.93 [−1.57, −0.29], *p* = 0.005; skin lesions: MD −1.19 [−2.28, 0.10], *p* = 0.03; arthralgia: MD −0.58 [−1.03, −0.13], *p* = 0.01 (6) RR 0.51 [0.28, 0.93], *p* = 0.03	n.r.
Li (2008) [21]	50/50 n.r. 1989 ISG	(A) HM (self-experience prescription, 2 times daily for 1 month, *n* = 25)	(B) Drug therapy (prednisone 10−30 mg, 1 time daily for 1 month, *n* = 25)	(1) Symptom score (2) Recurrence rate	(1) Oral ulcer: RR 1.00 [0.85, 1.17], NS; genital ulcer: RR 0.86 [0.54, 1.35], NS; eye inflammation: RR 0.88 [0.58, 1.33], NS; skin lesions: RR 0.83 [0.48, 1.44], NS; arthralgia: 0.96 [0.83, 1.10], NS (2) after 3 months of follow-up: RR 0.25 [0.06, 1.06], NS	Diarrhea (A: 4)
Zhou (2010) [22]	110/96 n.r. 1989 ISG	(A) HM (Ganzhi decoction, 2 times daily for 3 months, *n* = 50)	(B) Drug therapy (prednisone 10 mg, 1 time daily for 3 months, *n* = 46)	(1) Response rate (2) Recover rate (3) ESR (4) CRP (5) Symptom score	(1) RR 1.64 [1.28, 2.10], *p* < 0.0001 (2) RR 1.60 [0.63, 4.06], NS (3) MD −4.47 [−5.87, −3.07], *p* < 0.00001 (4) MD −3.68 [−4.95, −2.41], *p* < 0.00001 (5) Oral ulcer: MD −0.34 [−0.59, −0.09], *p* = 0.008; genital ulcer: MD −0.47 [−0.80, 0.14], *p* = 0.005; eye inflammation: MD −0.47 [−0.65, −0.29], *p* < 0.00001	n.r.
Wang (2012) [23]	30/30 A: 34.2; B: 40.0 A: 6.7; B: 8.5 1989 ISG; 1994 Standards TCM diagnosis criteria	(A) HM (PI prescription, 2 times daily for 2 months, *n* = 15)	(B) Drug therapy (thalidomide 50 mg, 1 time daily for 2 months, *n* = 15)	(1) Response rate (2) Recover rate (3) ESR (4) CRP (5) Symptom score (6) Recurrence rate	(1) RR 1.00 [0.83, 1.12], NS (2) RR 1.00 [0.24, 4.18], NS (3) MD 5.13 [−7.25, −17.51], NS (4) MD 5.43 [−6.05, 16.91], NS (5) Oral ulcer: MD 1.60 [−0.84, 4.04], NS; genital ulcer: MD 0.13 [−1.18, 1.44], NS; eye inflammation: MD −0.93 [−2.45, 0.59], NS; skin lesions: MD −0.27 [−2.70, 2.16], NS; arthralgia: MD 0.27 [−0.46, 1.00], NS (6) after 3 of months follow-up: RR 0.29 [0.07, 1.24], *p* = 0.03	Diarrhea (A: 2); insomnia (B: 11); dizziness (B: 11); constipation (B: 11)
Wang (2019) [24]	50/50 A: 39.1; B: 39.6 A: 7.5; B: 7.4 1989 ISG	(A) HM (modified Xiaoyao san, 2 times daily for 2 months, *n* = 25)	(B) Drug therapy (thalidomide 50 mg, 2 times daily for 2 months, *n* = 25)	(1) Response rate (2) ESR (3) CRP (4) Symptom score (5) Recurrence rate	(1) RR 1.38 [0.99, 1.91], NS (2) MD −11.40 [−11.99, −10.81], *p* < 0.0001 (3) MD −15.77 [−16.29, −15.25], *p* < 0.0001 (4) Oral ulcer: MD −0.80 [−0.94, −0.66], *p* < 0.0001; genital ulcer: MD −0.88 [−1.04, −0.72], *p* < 0.0001; eye inflammation: MD −0.93 [−2.45, 0.59], NS (5) after 12 months of follow-up: RR 0.29 [0.07, 1.24], NS	n.r.
Zhang (2015) [25]	100/100 A: 40.0; B: 36.2 A: 5.2; B: 3.7 2005 ICBD; 1994 Standards TCM diagnosis criteria	(A) HM (modified Gancao Xiexin decoction, 2 times daily for 3 months, *n* = 50)	(B) Drug therapy (prednisone 30 mg, 2 times daily; Thalidomide, 50 mg 1 time daily for 3 months, *n* = 50)	(1) Response rate (2) Recover rate (3) ESR (4) CRP (5) Symptom score	(1) RR 1.17 [0.94, 1.46], NS (2) RR 2.75 [0.94, 8.06], NS (3) MD −5.92 [−8.31, −3.53], *p* < 0.0001 (4) MD −5.71 [−7.73, −3.69], *p* < 0.0001 5) Oral ulcer: MD −0.34 [−0.59, −0.09], *p* = 0.01; genital ulcer: MD −0.43 [−0.72, −0.14], *p* = 0.004; eye inflammation: MD −0.38 [−0.53, −0.23], *p* < 0.00001; skin lesions: MD −2.24 [−2.42, −2.06], *p* < 0.0001; arthralgia: MD −0.12 [−0.24, 0.00], NS	n.r.
Yang (2013) [26]	180/180 32.8 2.5 1989 ISG	(A) HM (Bushen Huoxue Yuyang decoction, 2 times daily for 3 weeks, *n* = 90)	(B) Drug therapy (loxoprofen sodium 60 mg, 3 times dialy; Thalidomide 50 mg, 1 time daily for 3 weeks, *n* = 90)	(1) Response rate (2) Recover rate	(1) RR 1.17 [0.97, 1.40], NS (2) RR 2.24 [1.37, 3.65], *p* = 0.001	n.r.
Wu (2012) [27]	87/87 n.r. 1989 ISG	(A) HM (PI prescription, 2 times daily for 3 months, *n* = 49)	(B) Drug therapy (interferon a−2b injection, 3 times daily for 3 months, *n* = 38)	Response rate	RR 1.51 [1.01, 2.25], *p* = 0.05	n.r. detail (A: 1, B: 26)

BD: Behçet’s disease; CRP: C-reactive protein; ESR: erythrocyte sedimentation rate; HM: herbal medicine; ICBD: the International Criteria for Behçet’s Disease criteria; ISG: the International Study Group criteria; MD: mean difference; n.r.: not reported; PI: pattern identification; RR: risk ratio; TCM: traditional Chinese medicine.

**Table 2:** Composition of the herbal medicines for Behçet’s disease.

First Author (Year) [Ref]	Prescription	Consists of Herbs
Kao (2008) [20]	Yiqi Tuodu decoction	Astragali Radix 45 g, Paeoniae Radix Alba 30 g, Isatidis Folium 15 g, Glycyrrhizae Radix et Rhizoma 15 g, Glycyrrhizae Radix et Rhizoma Praeparata 15 g, Tripterygii Cortex 9 g, Angelicae Sinensis Radix 9 g, Angelicae Dauricae Radix 6 g
Li (2008) [21]	Modified Wenqin yin + Liuwei Dihuang wan	Atractylodis Rhizoma 30 g, Phragmitis Rhizoma 30 g, Sophorae Tonkinensis Radix et Rhizoma 10 g, Artemisiae Annuae Herba 30 g, Glycyrrhizae Radix et Rhizoma 20 g, Coptidis Rhizoma 10 g, Phellodendri Cortex 15 g, Scutellariae Radix 20 g, Ephedrae Herba 5 g, Paeoniae Radix Rubra 30 g, Coicis Semen 70 g, Alismatis Rhizoma 30 g, Dioscoreae Hypoglaucae Rhizoma 5 g
Zhou (2010) [22]	Ganzhi decoction	Scutellariae Radix 9 g, Coptidis Rhizoma 6 g, Pinelliae Rhizoma 15 g, Zingiberis Rhizoma 9 g, Vignae Semen 18 g, Angelicae Sinensis Radix 12 g, Atractylodis Macrocephalae Rhizoma 9 g, Atractylodis Rhizoma 12 g, Coicis Semen 15 g, Rehmanniae Radix 12 g, Gypsum Fibrosum 18 g, Cimicifugae Rhizoma 9 g, Glycyrrhizae Radix et Rhizoma 9 g
Wang (2012) [23]	(1) Modified Longdan Xiegan decoction (syndrome of toxic fire ablaze) (2) Modified Liangying Qingqi decoction (syndrome of fire-heat with ablaze, syndrome of toxic blazing of both qi and nutrient) (3) Modified Simiao Longan decoction (syndrome of dampness-heat accumulation) (4) Modified Zhibai Dihuang decoction (syndrome of dampness-heat and yin damage, syndrome deficiency fire with dampness) (5) Modified Xijiao Dihuang decoction (syndrome of toxic heat ablaze, syndrome of frenetic movement of blood due to heat)	(1) Glycyrrhizae Radix et Rhizoma 15 g, Glycyrrhizae Radix et Rhizoma Praeparata 15 g, Astragali Radix 30 g, Isatidis Folium 15 g, Smilacis Glabrae Rhizoma 30 g, Forsythiae Fructus 12 g, Conyzae Herba, Gardeniae Fructus, Scutellariae Radix, Akebiae Caulis, Alismatis Rhizoma, Plantaginis Semen, Bupleuri Radix, Glycyrrhizae Radix et Rhizoma, Angelicae Sinensis Radix, Rehmanniae Radix (2) Glycyrrhizae Radix et Rhizoma 15 g, Glycyrrhizae Radix et Rhizoma Praeparata 15 g, Astragali Radix 30 g, Isatidis Folium 15 g, Smilacis Glabrae Rhizoma 30 g, Forsythiae Fructus 12 g, Rhinocerotis Cornu, Dendrobii Herba, Gardeniae Fructus, Moutan Cortex, Rehmanniae Radix, Menthae Haplocalycis Herba, Coptidis Rhizoma, Paeoniae Radix Rubra, Scrophulariae Radix, Gypsum Fibrosum, Forsythiae Cortex, Lophatheri Herba, Phragmitis Rhizoma Hominis, Excrementum cum Aqua (3) Glycyrrhizae Radix et Rhizoma 15 g, Glycyrrhizae Radix et Rhizoma Praeparata 15 g, Astragali Radix 30 g, Isatidis Folium 15 g, Smilacis Glabrae Rhizoma 30 g, Forsythiae Fructus 12 g, Lonicerae Flos, Scrophulariae Radix, Angelicae Sinensis Radix (4) Glycyrrhizae Radix et Rhizoma 15 g, Glycyrrhizae Radix et Rhizoma Praeparata 15 g, Astragali Radix 30 g, Isatidis Folium 15 g, Smilacis Glabrae Rhizoma 30 g, Forsythiae Fructus 12 g, Dioscoreae Rhizoma, Moutan Cortex, Poria Sclerotium, Corni Fructus, Alismatis Rhizoma, Phellocendri Cortex, Rehmanniae Radix Praeparata, Anemarrhenae Rhizoma (5) Glycyrrhizae Radix et Rhizoma 15 g, Glycyrrhizae Radix et Rhizoma Praeparata 15 g, Astragali Radix 30 g, Isatidis Folium 15 g, Smilacis Glabrae Rhizoma 30 g, Forsythiae Fructus 12 g, Rhinocerotis Cornu, Rehmanniae Radix, Paeoniae Radix Alba, Moutan Cortex
Wang (2019) [24]	Modified Xiaoyao san	Bupleuri Radix 15 g, Angelicae Sinensis Radix 15 g, Paeoniae Radix Alba 15 g, Atractylodis Macrocephalae Rhizoma 15 g, Atractylodis Rhizoma 15 g, Moutan Cortex 15 g, Crataegi Fructus 15 g, Cyperi Rhizoma 15 g, Aurantii Fructus 15 g, Magnoliae Officinalis Cortex 15 g, Zingiberis Rhizoma Recens 15 g, Menthae Haplocalycis Herba 10 g, Glycyrrhizae Radix et Rhizoma 10 g Severe case: [Eye inflammation: Sophorae Flos 15 g, Chrysanthemi Flos 15 g, Dendrobii, Herba 20 g], [Genital ulcer: Sophorae Radix 10 g, Plantaginis Semen 20 g, Phellodendri Cortex 10 g], [Skin lesions: Moutan Cortex 15 g, Violae Herba 10 g, Taraxaci Herba 20 g], [Arthralgia: Dioscoreae Hypoglaucae Rhizoma 20 g, Siegesbeckiae Herba 30 g]
Zhang (2015) [25]	Modified Gancao Xiexin decoction	Glycyrrhizae Radix et Rhizoma 10 g, Scutellariae Radix 10 g, Codonopsis Pilosulae Radix 30 g, Zingiberis Rhizoma 10 g, Coptidis Rhizoma 6 g, Pinelliae Rhizoma 10 g, Astragali Radix 30 g, Angelicae Sinensis Radix 20 g, Paeoniae Radix Rubra 30 g, Phaseoli Semen 30 g, Cimicifugae Rhizoma 10 g, Jujubae Fructus 10 g
Yang (2013) [26]	Bushen Huoxue Yuyang decoction	Rehmanniae Radix Preparata, Lilii Bulbus, Angelicae Gigantis Radix, Anemarrhenae Rhizoma, Phellodendri Cortex, Scrophulariae Radix, Liriopis Tuber, Moutan Cortex, Paeoniae Radix Rubra, Tripterygii Cortex, Coicis Semen, Citri Reticulatae Pericarpium, Glycyrrhizae Radix et Rhizoma
Wu (2012) [27]	(1) Xuanhua Jiudu Yin (syndrome of dampness-heat accumulation, syndrome of toxic heat in the collaterals) (2) Erdong Runluo decoction (syndrome of yin deficiency in the collateral) (3) Buyang Tongluo decoction (syndrome of yang deficiency, syndrome of cold congealing in the collateral) (4) Shiwei Rongluo Yin (syndrome of tonify qi and replenish blood)	(1) Scrophulariae Radix 20 g, Lonicerae Flos 30 g, Bubali Cornu 10 g, Forsythiae Fructus 10 g, Angelicae Sinensis Radix 10 g, Taraxaci Herba 15 g, Paeoniae Radix Rubra 10 g, Atractylodis Rhizoma 10 g, Scutellariae Radix 6 g, Pinelliae Rhizoma 10 g, Phellodendri Cortex 10 g, Glycyrrhizae Radix et Rhizoma 10 g (2) Asparagi Radix 15 g, Liriopis Tuber 15 g, Rehmanniae Radix 10 g, Adenophorae Radix 15 g, Lycii Fructus 10 g, Angelicae Sinensis Radix 10 g, Anemarrhenae Rhizoma 10 g, Phellodendri Cortex 6 g, Paeoniae Radix Alba 15 g, Scrophulariae Radix 10 g, Moutan Cortex 10 g, Trionycis Carapax 15 g, Testudinis Carapax et Plastrum 15 g, Glycyrrhizae Radix et Rhizoma Praeparata 10 g (3) Cinnamomi Cortex 9 g, Zingiberis Rhizoma Praeparata 6 g, Rehmanniae Radix Preparata 20 g, Angelicae Sinensis Radix 10 g, Cervi Cornus Colla 10 g, Cinnamomi Ramulus 10 g, Sinapis Semen 6 g, Astragali Radix 15 g, Hirudo 3 g, Eupolyphaga Steleophaga 10 g, Glycyrrhizae Radix et Rhizoma 10 g (4) Asini Gelatinum 10 g, Polygoni Multiflori Radix 15 g, Codonopsis Pilosulae Radix 15 g, Atractylodis Rhizoma 10 g, Atractylodis Macrocephalae Rhizoma 10 g, Rehmanniae Radix Preparata 15 g, Cnidii Rhizoma10 g, Angelicae Sinensis Radix 10 g, Paeoniae Radix Alba 10 g

**Table 3:** Summary of findings.

Herbal Medicine for Behçet’s Diseases					
Patient or Population: Behçet’s Diseases Setting: Randomized Controlled Trials Intervention: Herbal Medicine Comparison: Drug Therapy					
Outcomes	No of Participants (Studies)	Certainty of the Evidence (GRADE)	Relative Effect (95% CI)	Anticipated Absolute Effects *	
				Risk with Drug Therapy	Risk Difference with Herbal Medicine
Response rate	573 (7 RCTs)	⨁◯◯◯ VERY LOW ^a,b,c^	RR 1.26 (1.09 to 1.45)	644 per 1000	167 more per 1000 (58 more to 290 more)
Recovery rate	655 (6 RCTs)	⨁⨁◯◯ LOW ^a,c^	RR 2.01 (1.39 to 2.91)	136 per 1000	138 fewer per 1000 (53 more to 260 more)
Recurrence rate	160 (4 RCTs)	⨁⨁◯◯ LOW ^a,c^	RR 0.40 (0.25 to 0.65)	160 per 1000	96 fewer per 1000 (387 fewer to 157 fewer)
ESR	306 (5 RCTs)	⨁◯◯◯ VERY LOW ^a,c,d^	**-**		MD 5.56 lower (9.99 lower to 1.12 lower)
CRP	306 (5 RCTs)	⨁◯◯◯ VERY LOW ^a,c,d^	-		MD 5.44 lower (12.73 lower to 1.86 higher)
Oral ulcers	306 (5 RCTs)	⨁◯◯◯ VERY LOW ^a,b,c^	-		MD 0.51 lower (0.85 lower to 0.16 lower)
Genital ulcers	306 (5 RCTs)	⨁◯◯◯ VERY LOW ^a,b,c^	-		MD 0.61 lower (0.91 lower to 0.31 lower)
Eye inflammation	306 (5 RCTs)	⨁◯◯◯ VERY LOW ^a,b,c^	-		MD 0.63 lower (0.93 lower to 0.34 lower)
Skin lesions	160 (3 RCTs)	⨁◯◯◯ VERY LOW ^a,b,c^	-		MD 1.62 lower (2.65 lower to 0.59 lower)
Arthralgia	160 (3 RCTs)	⨁◯◯◯ VERY LOW ^a,b,c^	-		MD 0.19 lower (0.56 lower to 0.17 higher)

CRP: C-reactive protein; CI: confidence interval; ESR: erythrocyte sedimentation rate; RR: risk ratio; MD: mean difference. * The risk in the intervention group (and its 95% confidence interval) is based on the assumed risk in the comparison group and the relative effect of the intervention (and its 95% CI). ^a^ Downgraded by one level: unclear or high risk of bias; ^b^ Downgraded by one level: heterogeneity is high; ^c^ downgraded by one level: small sample size; ^d^ downgraded by two levels: heterogeneity is very high. GRADE Working Group grades of evidence: low certainty (⨁⨁◯◯): our confidence in the effect estimate is limited: the true effect may be substantially different from the estimate of the effect; very low certainty (⨁◯◯◯): we have very little confidence in the effect estimate: the true effect is likely to be substantially different from the estimate of effect.

## Data Availability

Data is contained within the article.

## Supplementary Materials

The following are available online at https://www.mdpi.com/2072-6643/13/1/46/s1, Supplement 1. Search strategies.
