# Supplementary material for: Herbal Medicine for Behçet’s Disease: A Systematic Review and Meta-Analysis
Source: Nutrients. 2020 Dec 25;13(1):46. doi: 10.3390/nu13010046 (PMC7824385; doi:10.3390/nu13010046)
Supplement: Supplementary file 1 [file nutrients-13-00046-s001.pdf]

Supplement 1. Search strategies

1. PubMed

#1. “behcet syndrome” [mesh]

#2. “Behcet’s disease” or “Behcet syndrome” or “Adamantiades-Behcet’s Syndrome” or “Hulusi-behcet’s syndrome” or “Ouclo-bucco-genital syndrome” or “Touraine’s aphthosis” or “Triple Symptom Complex of Behcet” [tw]

#3. “Behcet’s disease” or “Behcet syndrome” or “Adamantiades-Behcet’s Syndrome” or “Hulusi-behcet’s syndrome” or “Ouclo-bucco-genital syndrome” or “Touraine’s aphthosis” or “Triple Symptom Complex of Behcet” [tiab]

#4. BD. ab.

#5. OR/ 1-4

#6. exp Medicine, Herbal/

#7. exp Plants, Medicinal/

#8. exp Medicine, Traditional/

#9. exp Drugs, Chinese Herbal/

#10. exp drugs, non-prescription/

#11. exp complementary therapies/

#12. exp Integrative Medicine

#13. ((traditional or chinese or herbal) adj (medic$ or preparation$ or drug$)).tw

#14. ((oriental or chinese) adj tradition$).tw.

#15. ((alternative or complementary) adj (therap$ or medicine$)).tw.

#16. OR/ 6-15

#17. 5 AND 16

2. EMBASE

#1. exp Behcet syndrome /

#2. “Behcet’s disease” or “Behcet syndrome” or “Adamantiades-Behcet’s Syndrome” or “Hulusi-behcet’s syndrome” or “Ouclo-bucco-genital syndrome” or “Touraine’s aphthosis” or “Triple Symptom Complex of Behcet”. tw.

#3. BD. ab.

#4. OR/1-3

#5. exp Medicine, Herbal/

#6. exp Plants, Medicinal/

#7. exp Medicine, Traditional/

#8. exp Drugs, Chinese Herbal/

#9. exp drugs, non-prescription/

#10. exp complementary therapies/

#11. ((traditional or chinese or herbal) adj (medic$ or preparation$ or drug$)).tw

#12. ((oriental or chinese) adj tradition$).tw.

#13. ((alternative or complementary) adj (therap$ or medicine$)).tw.

#14. exp Integrative Medicine

#15. OR/5-14

#16. 4 AND 15

3. Cochrane library

#1. "Behcet syndrome" [Mesh]

#2. Behcet's disease" or "Behcet syndrome" or "Adamantiades-Behcet's Syndrome" or "Hulusi-behcet's syndrome" or "Ouclo-bucco-genital syndrome" or "Touraine's aphthosis" or "Triple Symptom Complex of Behcet": ti, ab, kw

#3. BD. ab.

#4 OR/1-3

#5. "Medicine, Chinese Traditional" [Mesh]

#6. "Medicine, Kampo"[Mesh]

#7. "Medicine, Korean Traditional"[Mesh]

#8. Herbal or Decoction or herbal medicine or traditional medicine or TCM or tang: ti, ab, kw

#9 OR/5-8

#10 4 AND 9

4. CNKI, VIP, Wanfang

#1. 白塞氏病

#2. 狐惑病

#3. 白塞氏综合征

#4. 白塞综合征

#5. 白塞病

#6. 贝赫切特病

#7. 贝赫切特综合征

#8. 眼-口-生殖器三联综合征

#9. 皮肤 - 黏膜 - 眼综合征

#10. Behcet syndrome

#11. Behcet's syndrome

#12. Behcet's disease

#13. BD

#14. beh-CHETS

#15. Adamantiades-Behcet's Syndrome

#16. Hulusi-behcet's syndrome

#17. Touraine's aphthosis

#18.Triple Symptom Complex of Behcet

#19. OR/1-18

#20. 中药

#21. 中医

#22. 汤

#23. 中医药

#24. 方剂

#25. 方

#26. Chinese integrative medicine

#27. Herbal medicine

#28. Traditional Chinese medicine

#29. Traditional medicine

#30. Decoction

#31. TCM

#32. tang

#33. OR/20-32

#34. 19 AND 33

5. 5 Korean databases (OASIS, DBpia, RISS, KISS, and Korea Med)

#1. 베체트병

#2. 아다만티아데스-배채트 증후군

#3. 베체트 증후군

#4. 후루시-베체트 증후군

#5. 눈-볼-생식기 증후군

#6. 우렌 아프타증

#7. 베체트 삼중 복합 증후군

#8. Behcet

#9. OR/1-8

#10. 한약

#11. 처방

#12. 탕

#13. herbal

#14. decoction

#15. traditional medicine

#16. OR/10-15

#17. 9 AND 16
